# Weakly-coupled quasi-1D helical modes in disordered 3D topological insulator quantum wires

**DOI:** 10.1038/srep45276

**Published:** 2017-04-04

**Authors:** J. Dufouleur, L. Veyrat, B. Dassonneville, E. Xypakis, J. H. Bardarson, C. Nowka, S. Hampel, J. Schumann, B. Eichler, O. G. Schmidt, B. Büchner, R. Giraud

**Affiliations:** 1 Leibniz Institute for Solid State and Materials Research, IFW Dresden, D-01069 Dresden, Germany; 2 Max-Planck-Institut für Physik Komplexer Systeme, Nöthnitzer Straße 38, D-01187 Dresden, Germany; 3 Department of Physics, TU Dresden, D-01062 Dresden, Germany; 4 INAC-SPINTEC, Univ. Grenoble Alpes/CNRS/CEA, 17 Avenue des Martyrs, F-38054 Grenoble, France

## Abstract

Disorder remains a key limitation in the search for robust signatures of topological superconductivity in condensed matter. Whereas clean semiconducting quantum wires gave promising results discussed in terms of Majorana bound states, disorder makes the interpretation more complex. Quantum wires of 3D topological insulators offer a serious alternative due to their perfectly-transmitted mode. An important aspect to consider is the mixing of quasi-1D surface modes due to the strong degree of disorder typical for such materials. Here, we reveal that the energy broadening γ of such modes is much smaller than their energy spacing Δ, an unusual result for highly-disordered mesoscopic nanostructures. This is evidenced by non-universal conductance fluctuations in highly-doped and disordered Bi2Se3 and Bi_2_Te_3_ nanowires. Theory shows that such a unique behavior is specific to spin-helical Dirac fermions with strong quantum confinement, which retain ballistic properties over an unusually large energy scale due to their spin texture. Our result confirms their potential to investigate topological superconductivity without ambiguity despite strong disorder.

In a quantum wire of a strong 3D topological insulator (3DTI), the confinement of spin-helical Dirac fermions modifies the energy distribution of topological surface states in the transverse direction. Quasi-1D propagating surface modes form (see Fig. 1), and their transverse energy is quantized with an energy level spacing Δ = *hv*_*F*_/*L*_*p*_ that is directly related to the perimeter *L*_*p*_ of the nanostructure. In zero magnetic field, their energy spectrum is obtained from the intersection of the spin-textured Dirac cone with equally-spaced quantized planes, separated by *δk*_⊥_ = 2*π/L*_*p*_. A gap opens at the Dirac point and pairs of degenerate modes loose their topological protection. In presence of disorder, quantized modes are mixed and their transverse energy broadens over an energy scale Γ (see Fig. 1b). Besides, their energy spectrum can be tuned by applying a magnetic field *B*_||_ along the length of the nanowire^1^. Cyclic boundary conditions are changed and the additional phase introduced by the circulation of the vector potential along the perimeter, equivalent to a change in the transverse impulse, induces an overall energy shift of all modes. Importantly, a single gapless transverse mode with perfect transmission develops by tuning the magnetic flux Φ = *B*_||_ × *S* to half of a flux quantum Φ_0_ = *h/e, S* being the electrical cross section of the wire, and it is topologically protected^1^,^2^,^3^,^4^. This evolution between an even or an odd number of quantized modes is periodic in flux, with a period Φ_0_.

By proximity with a superconducting contact, this perfectly-transmitted mode induces topological superconductivity in the quantum wire and Majorana bound states are predicted to form at the S/TI interface^5^,^6^,^7^. A remarkable property is the robustness against non-magnetic disorder, in contrast to other systems hosting topological superconductivity^8^,^9^. In clean semiconducting nanowires, the interpretation of tunnel spectroscopy based on Majorana states^10^,^11^,^12^ is made complex by disorder^13^,^14^,^15^,^16^,^17^,^18^,^19^,^20^. In dirty 3DTI quantum wires, the influence of disorder is reduced and, interestingly, the nature of superconductivity could be tuned with the magnetic flux. Besides, the signature of Majorana states should appear in both the interface resistance of S/TI junctions^7^ and the current-phase relation of the supercurrent in Josephson junctions^21^. Although the experimental setup is rather simple, two important conditions must be satisfied so as to evidence zero-energy quasi-particles. First, the chemical potential must be tuned close to the Dirac point. This remains a challenge for all known 3D TI quantum wires, which are often strongly doped due to structural disorder. Charge compensation with extrinsic impurities is required, which increases the degree of disorder. Second, the broadening Γ of quantized transverse energies should remain smaller than their spacing Δ, a condition which is not obvious to realize in highly-disordered quantum wires. Here, we reveal that 3D TI nanostructures do satisfy the condition Γ < Δ despite strong disorder, due to the weak scattering of spin-helical modes, contrary to the case of metallic or semiconducting nanowires, for which the mixing of quantized modes by disorder is always efficient. This is unambiguously evidenced by disorder-sensitive quantum coherent transport experiments, which further give important information about the nature of surface charge transport. We report on the first observation of non-universal conductance fluctuations in a highly-disordered mesoscopic conductor, and show that the unusual transport properties of quasi-1D surface modes in 3D TI quantum wires are due to their spin structure.

In a mesoscopic wire, when the phase coherence length *L*_*φ*_(*T*) of quasi-particles becomes comparable to the length *L* of a conductor, quantum interference modifies the conductance. For a 3D TI quantum wire, the different quantum corrections to the conductance due to topological surface states can be separated, depending on the orientation of the applied magnetic field (see Supplementary Informations:sections I & III-B). Applying the field *B*_||_ parallel to the length of a nanostructure induces a well-defined Aharonov-Bohm phase and periodic oscillations of the conductance are induced by the magnetic flux Φ = *B*_||_ × *S*^1^. These were evidenced in Bi_2_Se_3_ nanostructures^22^, and a study of decoherence at very low temperature revealed the quasi-ballistic nature of charge transport in short-perimeter nanowires^23^. Applying a transverse magnetic field *B*_⊥_, disorder results in aperiodic conductance fluctuations, and their statistical properties depend on the nature and the dimensionality of the transport regime^24^. Whereas conductance fluctuations are sample-specific in ballistic conductors, provided that the classical dynamics of the conductor is not chaotic^25^,^26^, a small amount of impurities usually drives a system into the diffusive regime, for which conductance fluctuations are universal. In most cases, the ballistic-diffusive transition is rather abrupt since momentum scattering is strong enough that diffusive transport already occurs for a conductor length 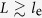, where *l*_e_ is the elastic mean free path, so that conductance fluctuations are usually universal in mesoscopic conductors^27^,^28^,^29^. In rare cases, for which momentum scattering is weak, this transition is smoother since it happens for 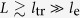, where the transport length *l*_tr_, typical of backscattered trajectories, becomes the relevant length scale for diffusive transport^24^. Such a situation happens for spin-helical Dirac fermions in a 3D topological insulator due to the spin-momentum locking, which results in enhanced forward scattering and therefore in a transport mean free path *l*_tr_ much longer than the elastic mean free path *l*_e_^30^,^31^. If a mesoscopic conductor is set within the ballistic-to-diffusive crossover, non-universal conductance fluctuations can be observed, and their statistical properties strongly depend on details of the quasi-particles’ energy spectrum and on their interplay with disorder, as shown below.

## Results

To investigate the nature of the *longitudinal* motion of helical Dirac fermions and the broadening of quantized surface modes by disorder, we study the conductance fluctuations of long mesoscopic 3D TI quantum wires in the limit *L* ≥ *l*_tr_, for which diffusive transport along the nanowire is expected. The Thouless energy remains smaller than the energy level spacing, in the sub-meV range. For clarity, we discuss below the results obtained in two different limits. First, large non-universal conductance fluctuations are evidenced in short-perimeter Bi_2_Se_3_ quantum wires (strong quantum confinement). In this limit *L*_*φ*_ ≫ *L*_*p*_, the periodic behavior of Aharonov-Bohm oscillations is not directly seen in magneto-conductance traces, due to their multiple-harmonic nature and disorder, but it can be revealed by a Fourier-transform analysis^23^. Due to the large energy level spacing Δ (reduced number of transverse modes), the non-universal behavior of conductance fluctuations is best observed. Second, smaller but clear non-universal conductance fluctuations are evidenced in long-perimeter Bi_2_Te_3_ quantum wires (small quantum confinement). In this limit 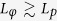, the periodic behavior of Aharonov-Bohm oscillations is directly seen in magneto-conductance traces (fundamental harmonic). Similar conclusions on the non-universal behavior of conductance fluctuations can be drawn, thus confirming their origin from surface mode transport, as well as the robust energy dependence of quasi-ballistic properties for all spin-helical modes.

## Experiments

Using a 3D-vector superconducting magnet, the quantum corrections to the classical conductance of mesoscopic 3D TI quantum wires were measured by controlling the longitudinal and transverse magnetic fields independently. For a complete *G(B*_||_, *B*_⊥_) mapping, the magnetic flux was set to a well-defined value (constant value of *B*_||_) and magneto-conductance traces *G(B*_⊥_) were measured. Importantly, the transverse magnetic field has little influence on the high-energy spectrum of confined Dirac fermions and it can therefore be conveniently used to probe aperiodic conductance fluctuations due to surface states, independently from Aharonov-Bohm conductance oscillations. See Supplementary Informations:sections I & II for details.

### Short-perimeter quantum wires

The largest amplitude of non-universal conductance fluctuations is revealed in short-perimeter Bi_2_Se_3_ quantum wires, with a perimeter *L*_p_ = 380 nm. Due to a high density of Se vacancies acting as donors, these nanostructures are metallic, with the surface Fermi energy *E*_*F*_ lying about 250 meV above the Dirac point. The total conductance results from a comparable contribution from surface and bulk carriers, as inferred from classical magneto-resistance and transconductance measurements on wide nanoribbons^31^,^32^, but conductance fluctuations in long wires are dominated by topological surface states, as shown below (see also Supplementary Informations: I). For helical Dirac fermions in the narrow Bi_2_Se_3_ wires considered here, the energy level spacing Δ ≈ 6 meV gives *N* ≈ 2*E*_*F*_/Δ ≈ 80 transverse modes, and we find *l*_tr_ < 300 nm, using *v*_F_ ≈ 5 · 10^5^ ms^−1^ obtained from angle-resolved photoemission spectroscopy^33^,^34^,^35^ (see Supplementary Informations: II-D).

**Figure 1:**
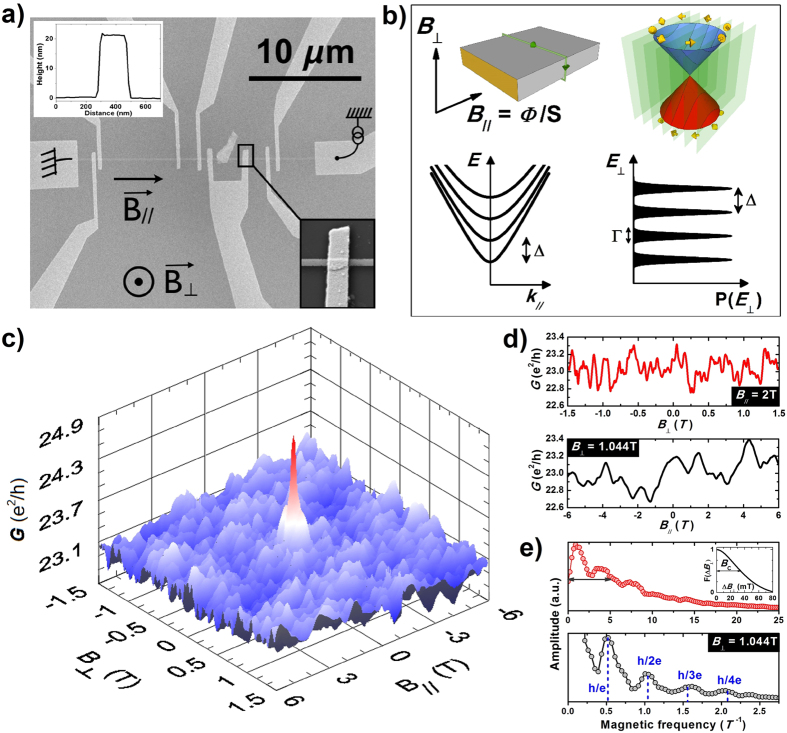
Quantum corrections to the classical conductance of a short-perimeter Bi_2_Se_3_ quantum wire. (**a**) Scanning electron microscope image of a narrow Bi_2_Se_3_ nanoribbon (width *w* = 170 nm, height *h* = 20 nm and perimeter *L*_p_ = 380 nm), contacted with CrAuAl leads. Three mesoscopic conductors are measured, with different lengths *L*_1_ ≈ 400 nm, *L*_2_ ≈ 1 *μ*m and *L*_3_ ≈ 6 *μ*m. Inset: Profile of the atomically flat structure measured by atomic force microscopy. (**b**) Schematics of the measurement geometry in magnetic field and of the quasi-1D energy modes induced by quantum confinement, with a transverse energy spacing Δ. Disorder leads to mode mixing, which results in the broadening Γ of quantized modes. (**c**) Longitudinal and transverse magneto-conductance *G(B*_||_, *B*_⊥_) of the quantum wire with length *L*_2_ = 1 *μ*m, for which *l*_tr_ ≈ *L*_p_ < *L*_2_ < *L*_*φ*_, measured at *T* = 30 mK. (**d**) Details from (**c**): *top, G(B*_⊥_) for a constant *B*_||_ = 2 T (aperiodic conductance fluctuations), and *bottom, G(B*_||_) for a constant *B*_⊥_ = 1.044 T (periodic Aharonov-Bohm oscillations). (**e**) Fast-Fourier transforms of magneto-conductance traces shown in (**d**). For conductance fluctuations (top), a small non-monotonous background is intrinsic to the limited field range studied and only the half-width at half-maximum (horizontal arrow) has a physical meaning (it corresponds to the 200 mT quasi-period seen in d). The overall damping over 25 *T*^−1^ relates to the 30 mT correlation field inferred from the correlation function *F*(Δ*B*_⊥_) = 〈*G(B*_⊥_)*G(B*_⊥_ + Δ*B*_⊥_)〉 shown in inset. For Aharonov-Bohm oscillations (bottom), four harmonics clearly arise from the background (vertical dashed lines).

**Figure 2:**
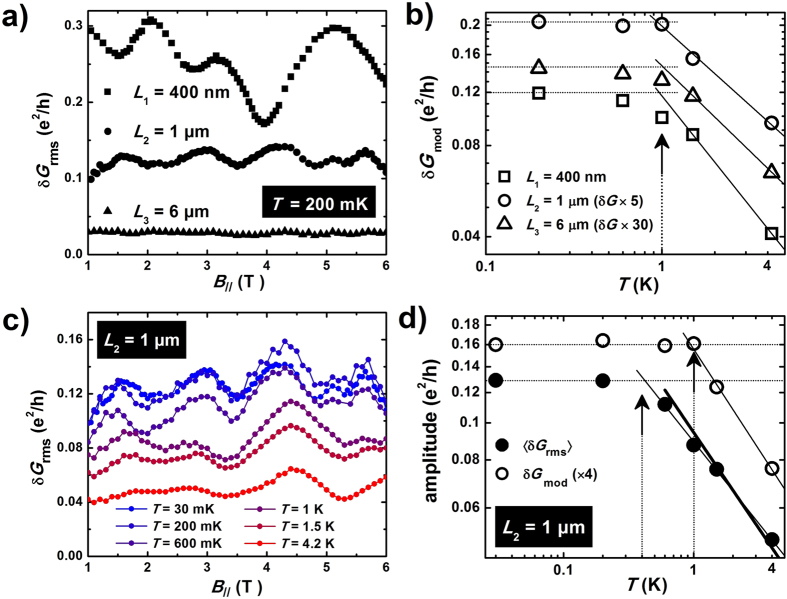
Non-universal conductance fluctuations modulated by the longitudinal field. (**a**) Longitudinal-field dependence of the standard deviation of conductance fluctuations *δG*_rms_ measured at *T* = 200 mK for three different lengths, with *l*_tr_ ≈ *L*_1_ < *L*_*φ*_, *l*_tr_ < *L*_2_ ≈ *L*_*φ*_ and *l*_tr_ < *L*_*φ*_ < *L*_3_. For a given value of the magnetic flux, *δG*_rms_ is evaluated from *B*_⊥_ sweeps over a 3T range, with no need of a background substraction (negligible classical magneto-resistance). Non-universal conductance fluctuations due to surface modes result in a Φ_0_-periodic modulation of *δG*_rms_, corresponding to a 1.6 T period in *B*_||_. (**b**) Temperature dependence of the modulation 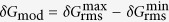 for all three lengths measured. Dotted lines correspond to the saturation values 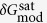 at low temperatures and solid lines to a power-law behavior at high temperatures. The arrow shows a single crossover temperature T^*^ = 1 K ± 100 mK. (**c**) Longitudinal-field dependence of *δG*_rms_ for the intermediate length *L*_2_ = 1 *μ*m, with 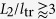, measured at different temperatures. (**d**) Temperature dependence of the amplitude of the standard deviation averaged over *B*_||_, 〈*δG*_rms_〉, and of its maximum peak-to-peak modulation *δG*_mod_ by a longitudinal field, as inferred from (**c**). Dotted lines correspond to the saturation regimes at low temperatures and solid lines to a power-law behavior at high temperatures. Arrows indicate the different crossover temperatures. Thick line: 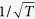 dependence of *δG*_rms_(*T*) in the regime *L*_*φ*_(*T*) < *L*_2_.

An example of the full mapping of quantum corrections to the classical conductance, measured at *T* = 30 mK, is shown in Fig. 1c) for a wire length *L*_2_ = 1 *μ*m. Quantum interference of topological surface states results in a sharp weak anti-localization peak around zero field and in a different field dependence of the conductance depending on whether the magnetic field is swept along the wire axis (slow Aharonov-Bohm oscillations) or perpendicular to it (fast aperiodic fluctuations), as seen in Fig. 1d). The distinct nature of quantum interference patterns in finite field is clearly evidenced by the Fourier transform of the magneto-conductance traces measured in either a longitudinal or a perpendicular field (Fig. 1e), revealing their periodic or aperiodic characteristics, respectively, and their very different correlation fields. To investigate the nature of conductance fluctuations, the standard deviation *δG*_rms_ is evaluated from *B*_⊥_ sweeps over a 3T range, which is much larger than the correlation field *B*_C_ ≈ 30 mT inferred from the correlation function 〈*G(B)G(B* + Δ*B*)〉 (see inset in Fig. 1e), for constant values of the magnetic flux (*B*_||_ is fixed). At low-enough temperatures, when *L*_*φ*_(*T*) ≥ *L, δG*_rms_ saturates at a value 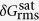, as known for a mesoscopic conductor in the fully-coherent regime. However, this value varies with the longitudinal magnetic field (Fig. 2a) instead of being fixed to a universal value, as expected for diffusive transport (*L*_*φ*_ ≥ *L* ≥ *l*_tr_) in general and for highly-disordered topological insulators without quantum confinement in particular^36^,^37^,^38^. The dominant contribution of spin-helical quantized modes to such non-universal conductance fluctuations is unambiguously confirmed by their flux dependence. Indeed, a striking feature is the *periodic modulation* of *δG*_rms_(*B*_||_), a hallmark of surface-state transport. All wires show a 1.6 T period in *B*_||_, which corresponds to a Φ_0_ periodicity in flux, in agreement with the electrical cross-section of surface states, taking surface oxidation into account^23^. Remarkably, the amplitude of the modulation is reduced with increasing the length *L* (Fig. 2a) whereas its damping with temperature does not depend on *L* (Fig. 2b). Note also that these non-universal conductance fluctuations remain visible even in the longest conductor studied with *L* = 6 *μ*m (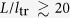), due to quasi-ballistic transport. It could be tempting to relate this behavior to a periodic topological transition due to the perfectly-transmitted mode, as considered in recent studies of Aharonov-Bohm oscillations in 3D TI quantum wires^39^,^40^,^41^, but this is not the case here and we show below that non-universal conductance fluctuations result from multi-mode transport and are actually the signature of the weak mixing between spin-helical quantized modes by disorder, due to their spin texture.

The analysis of the temperature dependence of the standard deviation *δG*_rms_ and of its modulation 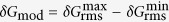 by a magnetic flux further reveals two important properties, which are the diffusive nature of the longitudinal motion and the strength of the disorder-induced broadening of quantized transverse modes. We consider the conductor with length *L*_2_ = 1 *μ*m (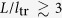) with more details (see Fig. 2c,d). Since the phase coherence length of helical Dirac fermions is comparable to or longer than the transverse dimension of the nanowire, quantum coherent transport occurs in the quasi-1D limit over the full temperature range studied. At high-enough temperature, *L*_*φ*_(*T*) < *L* and the size averaging of conductance fluctuations varies as 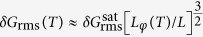 (see refs 24 and 29). In the 1D diffusive regime and for decoherence induced by electron-electron interactions^42^, 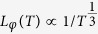 gives 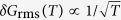. This reasonably agrees with our measurements for both *δG*_rms_ and its flux average 〈*δG*_rms_〉 (see Fig. 2d), which confirms the different nature of charge transport for the longitudinal and transverse motions in our quantum wires, being diffusive or ballistic, respectively. At very low temperature, *δG*_rms_ saturates when *L*_*φ*_(*T*) ≥ *L*, a crossover that indeed occurs at *T* ≈ 200 mK for the 1 *μ*m long conductor, in agreement with our previous estimations of *L*_*φ*_ inferred from Aharonov-Bohm oscillations^23^. Strikingly, the temperature crossover is much higher for the modulation of *δG*_rms_ with the longitudinal field. *δG*_mod_ is temperature independent up to about T^*^ = 1 K, a behavior found for all wire lengths (see Fig. 2b). This shows that the amplitude of non-universal conductance fluctuations is not directly related to *L*_*φ*_, a result which is not surprising since the flux modulation of *δG*_rms_ depends on the length even in the fully-coherent regime (as seen in Fig. 2a). This modulation indeed depends on details of the average evolution of quantum states in the reciprocal space, for which the relevant length scale is the transport length, related to the disorder broadening of energy levels. Therefore, the crossover observed at T^*^ rather corresponds to the limit when the thermal broadening of quantized levels compares to their disorder broadening (see Supplementary Informations: III-C). Thus, this measure gives a direct access to the strength of disorder broadening, and we infer Γ ≈ 4 × k_B_T^*^ ≈ 0.4 meV. This value is much smaller than the energy level spacing Δ ≈ 6 meV, so that the mixing of transverse modes by disorder remains limited to a few conductance channels close in energy, an important finding that is confirmed below by theory.

### Long-perimeter quantum wires

**Figure 3:**
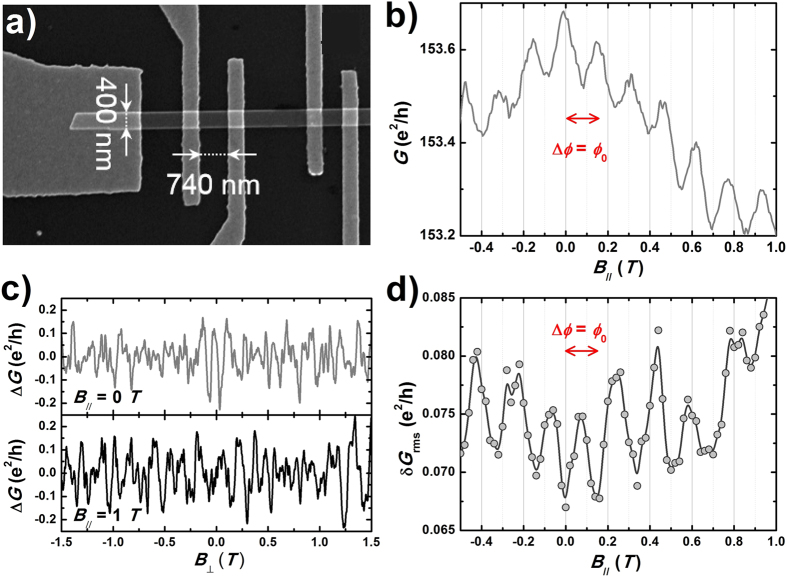
Non-universal conductance fluctuations in a long-perimeter Bi_2_Te_3_ quantum wire. (**a**) Scanning electron microscope image of a Bi_2_Te_3_ nanoribbon (width *w* = 400 nm, height *h* = 70 nm and perimeter *L*_p_ = 940 nm), contacted with CrAu contact pairs with three different lengths *L*_1_ ≈ 740 nm, *L*_2_ ≈ 1.6 *μ*m and *L*_3_ ≈ 3.6 *μ*m. (**b**) Longitudinal magneto-conductance *G(B*_||_) for the wire with length *L*_1_ = 740 nm (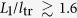), measured at *T* = 100 mK, showing periodic Aharonov-Bohm oscillations with a 150 mT period. (**c**) Perpendicular magneto-conductance *G(B*_⊥_) at *T* = 100 mK, after the substraction of a slowly-varying background, showing aperiodic conductance fluctuations with a 16 mT correlation field. (**d**) Longitudinal-field dependence of *δG*_rms_, inferred from *G(B*_⊥_) traces measured in the ±1.5 T range. The solid line is the B-spline fit associated to the data points. The modulation corresponds to the expected Aharonov-Bohm Φ_0_ flux period, shown as red arrows.

To confirm the results obtained in a short-perimeter Bi_2_Se_3_ nanowire, we studied the quantum conductance fluctuations in a wider nanoribbon of another 3D topological insulator Bi_2_Te_3_ with a perimeter *L*_p_ = 940 nm (Fig. 3), also grown by vapor transport, for three pairs of CrAu contacts (*L*_1_ ≈ 740 nm, *L*_2_ ≈ 1.6 *μ*m and *L*_3_ ≈ 3.6 *μ*m). The confinement energy Δ ≈ 1.6 meV is reduced, and the larger electrical cross section gives a shorter AB period Δ*B*_||_ = 150 mT. Assuming a Fermi energy *E*_*F*_ ≈ 120 meV typical for such Bi_2_Te_3_ nanostructures^41^, we obtain an upper bound for the transport length *l*_tr_ < 450 nm from the conductance, so that the condition *L* > *l*_tr_ is satisfied for all mesoscopic conductors, and *N* ≈ 150 transverse modes. Due to the dominant contribution of the fundamental h/e harmonic (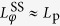) and to an AB period smaller than the quasi-period of conductance fluctuations due to (*L*_*ϕ*_^BS^ < *L*_p_/*π*), periodic Aharonov-Bohm oscillations are directly visible in the flux dependence of the conductance, as shown in Fig. 3b for the shorter length *L*_1_ (see also Supplementary Informations: II-A).

Magneto-conductance traces are very different if the magnetic field is applied perpendicularly to the nanoribbon, and reproducible conductance fluctuations are evidenced (see Fig. 3c, for two different values of the magnetic flux Φ = *B*_||_ × *S*). Their correlation field is small (*B*_C_ ≈ 16 mT), so that accurate values of the conductance standard deviation can be extracted from *G(B*_⊥_) traces over a ±1.5 T range. The Φ_0_-periodic modulation of *δG*_rms_(*B*_||_) is unambiguously evidenced in Fig. 3d, a result confirmed for the other two longer conductors (see Supplementary Informations: II-C). As found for Bi_2_Se_3_ quantum wires, the relative change in *δG*_rms_ is much larger than the conductance change due to the AB effect. Note also that there is no correlation between the conductance and the amplitude of conductance fluctuations (a thorough analysis in given in Supplementary Informations: II-D), which can be readily seen in Fig. 3b,d), where a minimum of conductance can give either a maximum or a minimum in *δG*_rms_. This clear confirmation of non-universal conductance fluctuations due to topological surface states in a Bi_2_Te_3_ nanoribbon shows that the weak coupling of spin-helical surface modes is a general property of quasi-ballistic 3D topological insulator quantum wires.

## Theory

**Figure 4:**
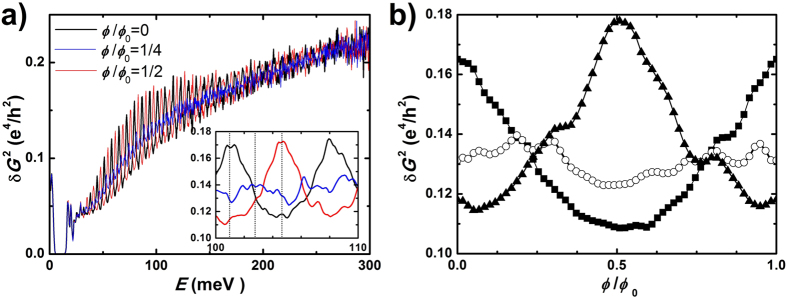
Calculation of the conductance fluctuations in a disordered 3D TI nanostructure. (**a**) Energy dependence of the conductance variance in a disordered 3D topological insulator quantum wire with transverse dimensions similar to the experiment (width *w* = 120 nm, height *h* = 20 nm), a length *L* = 350 nm and a correlation length of disorder of 10 nm, typical of disordered Bi_2_Se_3_. Calculations are done for a constant transverse field *B*_⊥_ = 1 T and three different longitudinal fields which correspond to a magnetic flux Φ = *n*Φ_0_ 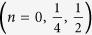. The inset shows a zoom in a reduced energy window. (**b**) Flux dependence of the conductance variance for three different energies shown as dotted lines in a): *E*_0_ = 101 meV (■), 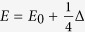 (○) and 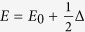 (▲), with 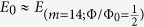 and Δ = 7.2 meV. When a conductance channel opens, non-universal conductance fluctuations are modulated by the magnetic flux.

Based on scattering matrix formalism, we show that this unusual behavior in mesoscopic transport results from a combination of both the even energy spectrum of spin-helical Dirac fermions under periodic boundary conditions and their enhanced forward scattering by disorder, due to their spin helicity. To theoretically model our experiments we adapt a continuous Dirac fermion description of the surface state^1^,^43^ and take the bulk as an inert insulator (see Supplementary Information-section III-A). For a fixed chemical potential *μ*, the number of propagating modes in the nanowire is *N* = 2*μ*/Δ. Since in our experiments *N* is generally large due to the pinning of the Fermi energy far away from the Dirac point, we studied the evolution of conductance fluctuations over a broad energy range, and our results reveal that the ballistic nature of quantized modes persists in the large-*N* limit. The statistics of conductance fluctuations is obtained from sampling over many different microscopic configurations of disorder (~1000), and the conductance variance 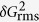 is calculated for three different values of the flux 
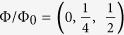
, which correspond to different configurations of the quantized energy spectrum. For 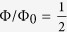, *E*_*m*_ = *m*Δ, where *m* is an integer (mode index). As shown in Fig. 4a) for a fixed perpendicular field *B*_⊥_ = 1 T, we evidence the energy dependence of the conductance variance, revealing non-universal conductance fluctuations. Close to the charge-neutral Dirac point (*E* = 0), the physics is dominated by a chiral mode arising from quantum Hall physics^7^,^44^,^45^. As the energy is increased, quantized transverse modes are weakly perturbed by the transverse field and a clear oscillating behavior of the conductance variance is observed, which corresponds to the opening of discrete transport channels. Since the exact value of the chemical potential at which new channels open depends on the parallel field, these oscillations are shifted by the flux. As a consequence, non-universal conductance fluctuations can be probed at a fixed energy by measuring the flux dependence of the conductance variance. This is shown in Fig. 4b) for three different energies, which correspond to either the opening of a conductance channel (*E* = 101 meV and *E* = 104.6 meV) or to nearly closed or opened channels (*E* = 102.8 meV). The modulation of the variance is indeed periodic in flux, with a period Φ_0_, and harmonics are clearly present, as expected in the fully coherent regime. Importantly, the amplitude of the modulation can be nearly as large as the variance itself, and this is a direct consequence of the limited mixing of quasi-1D modes by disorder. At larger energies, the modulation is reduced due to a larger number of modes (~3–4) contributing to conductance fluctuations, but it remains significant even at *E* = 250 meV, for which both the conductance variance and the amplitude of its modulation compare reasonably well with our experiments.

## Discussion

The existence of non-universal conductance fluctuations in a diffusive conductor is at odds with standard theories for massive quasi-particles^27^,^28^,^29^ and for Dirac fermions^46^,^47^, which predict universal values of *δG*_rms_ in the fully-coherent metallic regime (*N* ≫ 1). Similar results were found for Dirac fermions in presence of a strong long-range disorder and without quantum confinement, and deviations from a universal value were only found near the ballistic regime^48^. In presence of quantum confinement, although reduced or enhanced conductance fluctuations can occur for a ballistic conductor with a small number of quantized modes^49^,^50^,^51^,^52^,^53^, universality is restored even by a small disorder if the number of transverse modes exceeds a few units^54^. In the long Bi_2_Se_3_ and Bi_2_Te_3_ quantum wires studied here, both conditions *L*_*φ*_ > *L* ≥ *l*_tr_ ≫ *l*_*e*_ and *G* ≫ *e*^2^/*h* (number of populated transverse modes *N* ≫ 1) should therefore set the system in the universal regime. This is clearly not the case, due to the anisotropic scattering of spin-helical Dirac fermions, and our results suggest that the ballistic nature of quantized transverse modes is robust against disorder, even when approaching the diffusive limit.

In this work, we argue that non-universal conductance fluctuations find their origin in the small broadening Γ of transverse energy levels relative to their energy spacing Δ, as a result of the weak coupling of quasi-1D helical modes by disorder. In general, Γ ≫ Δ so that all energy levels overlap. All transverse modes are mixed together by disorder, so that the discretization is washed out and conductance fluctuations are universal. In this limit, there would be no difference between a topological insulator and a charge-accumulation layer at the surface of a semiconductor with strong spin-orbit coupling. This regime corresponds to the case of both metallic and semiconducting nanowires, for which Δ is small and a small disorder induces a rather large broadening Γ of quantized energy levels. In the opposite limit considered here, Γ < Δ, a specific transport regime emerges in the crossover between ballistic and diffusive transport, for which signatures of the discreteness and the Dirac nature of the spectrum can be probed even in presence of a large number of modes and close to the diffusive regime for the longitudinal motion. In disordered 3D topological insulator nanostructures, this transport regime is surprisingly robust against a high density of scatterers and it exits over a significantly large energy range. This is due to the weak scattering of spin helical Dirac fermions by disorder, which limits disorder broadening, as well as to the increased energy quantization of Dirac fermions, with respect to massive quasi-particles, and the regular distribution of quantized energies under cyclic boundary conditions. A higher degree of disorder than the one considered here (*l*_e_ ≈ 30 nm) would be required to achieve diffusive transport for high-energy transverse modes. Even in this case, transport would nevertheless remain ballistic close to the Dirac point due to the perfectly-transmitted mode^4^. This situation of *pseudo-ballistic* transport in a diffusive conductor corresponds to the counterpart of *pseudo-diffusive* transport found in ballistic graphene nanostructures at the Dirac point^55^,^56^,^57^,^58^.

Despite strong disorder, our finding of a weak coupling between quasi-1D helical modes is confirming the potential of 3D TI quantum wires to investigate topological superconductivity. Contrary to the case of semiconducting quantum wires proximitized by a superconducting contact^15^, the limited degree of mode mixing could make the detection of a zero-energy Majorana mode by tunnel spectroscopy more difficult but, importantly, novel measurement schemes can be considered^7^,^21^. Beyond the field of topological superconductivity, our finding also shows that new opportunities in mesoscopic physics can be considered, for instance to investigate the crossover between ballistic and diffusive transport, which was only studied for a relatively weak disorder^59^,^60^ and remains overlooked in a regime of strong disorder. Also, it confirms the possibility to investigate the physics of 3D TI quantum wires with a small number of spin-textured modes, for which a fractional quantization of the conductance and its fluctuations induced by disorder in zero field were predicted^61^, as well as the formation of chiral states under a transverse magnetic field^7^,^61^.

## Methods

### Growth of Bi_2_Se_3_ and Bi_2_Te_3_ quantum wires, and Sample preparation

The synthesis of single-crystalline Bi_2_Se_3_ and Bi_2_Te_3_ nanostructures was realized by catalyst-free vapor transport in a vacuum sealed silica tube with two compartments separated by a bottleneck^62^. One chamber contains a Bi_2_Se_3_ or Bi_2_Te_3_ powder (Sigma-Aldrich) and the other a *p*^++^-Si/SiO_*x*_ substrate. Electrical properties of wide ribbons were studied in high magnetic fields^32^ or using an electrical back gate^31^. Individual nanostructures were imaged with a scanning electron microscope, and their height was measured by atomic force microscopy. For ohmic contacts, a lift-off of CrAuAl or CrAu was made after electron-beam lithography (Zeiss NVision 40), Ar-ion milling and metal deposition (Plassys).

### Magneto-transport measurements

Quantum corrections to the conductance of 3D topological insulator quantum wires were measured in a four-probe geometry with lock-in amplifiers, from 4.2 K down to the 20 mK base temperature of a ^3^He/^4^He dilution refrigerator (Oxford Kelvinox 300), with a small-enough current polarisation to avoid electronic heating. The base electronic temperature is smaller than 50 mK^23^. Using a three-dimensional vector magnet (Oxford Instruments), the magneto-conductance was investigated with the magnetic field applied either along the nanowire axis, *B*_||_ up to 6T, or perpendicular to the sample plane, *B*_⊥_ up to 2T, with an accuracy of alignement better than 1°.

## Additional Information

**How to cite this article:** Dufouleur, J. *et al*. Weakly-coupled quasi-1D helical modes in disordered 3D topological insulator quantum wires. *Sci. Rep.* **7**, 45276; doi: 10.1038/srep45276 (2017).

**Publisher's note:** Springer Nature remains neutral with regard to jurisdictional claims in published maps and institutional affiliations.

## Supplementary Material

Supplementary Information
