# Supplementary material for: Weakly-coupled quasi-1D helical modes in disordered 3D topological insulator quantum wires
Source: Sci Rep. 2017 Apr 4;7:45276. doi: 10.1038/srep45276 (PMC5379752; doi:10.1038/srep45276)
Supplement: Supplementary Information [file srep45276-s1.pdf]

Weakly-coupled quasi-1D helical modes in disordered 3D topological insulator quantum wires

J. Dufouleur,[1] L. Veyrat,[1] B. Dassonneville,[1] E. Xypakis,[2] J. H. Bardarson,[2] C. Nowka,[1] S. Hampel,[1] J. Schumann,[1] B. Eichler,[1] O. G. Schmidt,[1] B. Büchner,[1,3] and R. Giraud[1,4]

[1]*Leibniz Institute for Solid State and Materials Research, IFW Dresden, D-01069 Dresden, Germany*
[2]*Max-Planck-Institut für Physik Komplexer Systeme, Nöthnitzer Straße 38, D-01187 Dresden, Germany*
[3]*Department of Physics, TU Dresden, D-01062 Dresden, Germany*
[4]*INAC-SPINTEC, Univ. Grenoble Alpes/CNRS/CEA, 17 Avenue des Martyrs, F-38054 Grenoble, France*

I. CONDUCTANCE FLUCTUATIONS AND AHARONOV-BOHM OSCILLATIONS

Different quantum corrections to the conductance of a 3D topological insulator nanostructure can be studied in a finite magnetic field, the direction of which determines the exact nature of the quantum interference probed. In the diffusive regime, such a difference is best captured by a semi-classical approach[1], considering all possible closed-loop trajectories (see Fig. 1). If the magnetic flux is trapped within closed loops with a well defined area (such as the transverse cross section for topological surface states -see Fig. 1:bottom,left), the conductance can show periodic oscillations with the magnetic field and their period only depends on the geometry of the nanostructure. If, instead, there is a size distribution of closed loops (such as for all other cases considered in Fig. 1), the conductance can show reproducible aperiodic fluctuations with the magnetic field and their quasi-period will depend on the longest quantum coherent paths within the plane perpendicular to the applied field, which are related to either the phase-coherence length or the geometry of the conductor, or to both of them. This intuitive description remains qualitatively relevant for a ballistic conductor and the nature of quantum corrections to the conductance depends on the size statistics of classical closed-loop trajectories (and therefore on the geometry of the conductor), a property related to the level statistics in the energy spectrum of quantum states[2].

Importantly, the relative contribution of bulk carriers and topological surface states to the amplitude of quantum conductance fluctuations strongly depends on both the nature of charge transport (ballistic/diffusive), related to the transport length $l_{\rm tr}$, and the dimensionality of coherent transport, related to the phase coherence length L_φ. For a 3D topological insulator with a typically strong disorder (mean-free path of about 30 nm), charge transport is 3D for bulk states ($l_{\rm tr}^{\rm BS} \approx l_{\rm e} < \{w, h\}$), whereas it can be 2D ($L_{\rm p} \gg l_{\rm tr}^{\rm SS}$) or 1D ($L_{\rm p} \lesssim l_{\rm tr}^{\rm SS}$) for topological surface states (with $l_{\rm tr}^{\rm SS} \gg l_{\rm tr}^{\rm BS}$). Due to a different phase coherence length, quantum coherent transport is 1D over a wide temperature range for surface states ($L_\varphi^{\rm SS} > \{w, h\}$) whereas it is 3D for bulk states ($L_\varphi^{\rm BS} < \{w, h\}$), but at very low temperatures where it becomes 1D when $L_\varphi^{\rm BS} > \{w, h\}$. Since the phase coherence lengths depend on temperature and since the decoherence mechanism can be different for bulk and surface states with quantum confinement, their relative contribution to conductance fluctuations depends on both the length of the mesoscopic conductor and temperature.

As a quantitative example at T =200mK, the phase coherence lengths are $L_\varphi^{\rm SS} \approx 1$ μm and $L_\varphi^{\rm BS} \approx$390nm. The latter value is inferred from measurements on a wide Bi_2Se_3 nanoribbon with similar bulk-transport properties[3], assuming that decoherence is limited by electron-electron interactions. Besides, it is in good agreement with the quantitative values found here for the finite contribution to $\delta G_{\rm rms}$ due to bulk states, giving a shift of its average $< \delta G_{\rm rms} >$ over $B_\parallel$, which is best visible at low temperature and for a short wire. Since quantum coherent transport is 1D for both bulk and surface states, the self averaging of $\delta G_{\rm rms}$ varies as $(L_\varphi/L)^{\frac{3}{2}}$, and we find a relative contribution of bulk carriers of about 16% for $L_2 = 1$ μm and 50% for $L_1 =$ 400nm. Most importantly, this bulk contribution to conductance fluctuations is universal (diffusive transport). Therefore, its standard deviation does not depend on the magnetic flux, contrary to the contribution of topological surface states in the quasi-ballistic regime. The latter gives rise to non-universal conductance fluctuations with a flux-periodic modulation of $\delta G_{\rm rms}$. This modulation is found to be as large as $\delta G_{\rm rms}$ if $L \approx l_{\rm tr}^{\rm SS}$ or reduced in the ratio $l_{\rm tr}^{\rm SS}/L$ if $L > l_{\rm tr}^{\rm SS}$, in very good quantitative agreement with the results shown in Fig.2a) in the main text and $l_{\rm tr}^{\rm SS} \approx 300$ nm.

Below, we consider the different contributions to quantum interference in a 3D topological insulator nanowire, probed by sweeping the magnetic field applied either along or perpendicular to the nanostructure.

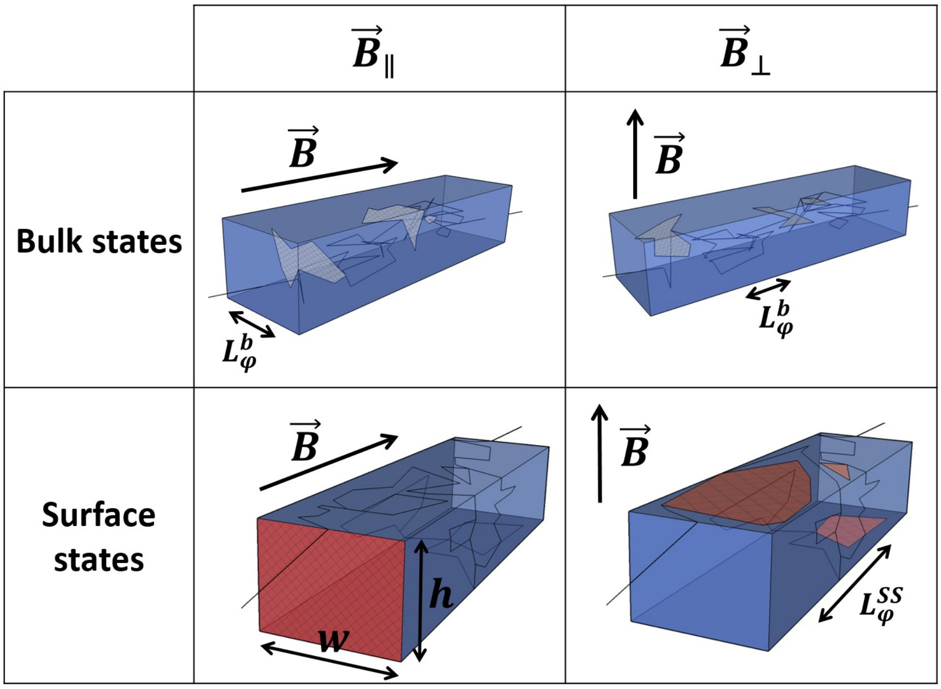

Figure 1: **Quantum interference in a 3D topological insulator nanostructure. Top**, Bulk carriers only contribute to universal conductance fluctuations, for both cases of a longitudinal field (left) and a perpendicular field (right). In the case considered here, $L_\varphi^{\mathrm{BS}} < \{w, h\}$ so that the correlation field is given by $B_{\mathrm{C}}^{\mathrm{BS}} = \Phi_0/(L_\varphi^{\mathrm{BS}})^2$; **Bottom**, Topological surface states only contribute to periodic quantum oscillations for the case of a longitudinal field (left), with a period $\Delta B_\parallel = \Phi_0/(w \times h)$ (Aharonov-Bohm) or $\Delta B_\parallel = \frac{1}{2}\Phi_0/(w \times h)$ (Altshuler-Aronov-Spivak), whereas they only contribute to non-universal conductance fluctuations when a perpendicular field is applied (right), with a correlation field given by $B_{\mathrm{C}}^{\mathrm{SS}} = \Phi_0/(L_\varphi^{\mathrm{SS}} \times w)$.

A. Influence of a longitudinal field

Applying a longitudinal field $B_\parallel$, bulk states only induce universal conductance fluctuations, whereas topological surface states do not give any aperiodic conductance fluctuations, since there is no flux trapped by surface closed loops, and they only result in periodic Aharonov-Bohm oscillations of the conductance, even at rather high temperatures (long phase coherence length L_φ^{SS}, with respect to the perimeter L_{p}).

As sketched in Fig. 1, the different quantum corrections to the conductance are:

- Aperiodic *universal* conductance fluctuations from bulk states. Since in general $L_\varphi^{\mathrm{BS}} < L$, their amplitude has a power-law dependence with $L_\varphi^{\mathrm{BS}}/L$ (see ref.[1] for details), and their correlation field is given by $B_{\mathrm{C}}^{\mathrm{BS}} = \Phi_0/(\max\{L_\varphi^{\mathrm{BS}}), w\} \times \max\{L_\varphi^{\mathrm{BS}}), h\}$.
- Periodic Aharonov-Bohm (AB, h/e flux periodicity) or Altshuler-Aronov-Spivak (AAS, $h/2e$ flux periodicity) oscillations from surface states, with a period $\Delta B_\parallel = \Phi_0/S$ (or $\Phi_0/2S$) related to the transverse cross section $S = w \times h$. Such oscillations exist both in the ballistic regime and in the diffusive regime, also for rather long mesoscopic conductors due to the weak scattering by disorder.

  i) In the diffusive regime, their amplitude has an exponential dependence with L_φ^{SS}. For long mesoscopic conductors, with $L \gg L_\varphi^{\mathrm{SS}} > L_{\mathrm{p}}$, only AAS oscillations should survive whereas AB oscillations are damped by ensemble averaging over uncorrelated coherent segments. However, this reduction is relatively small due to the large enhancement of the transport length for topological surface states.

  ii) In the ballistic regime, the amplitude of both AB and AAS oscillations depends on the transmission of transverse modes and their energy spectrum, which is periodically modified by the flux, in addition to the phase coherence length. Importantly, quantum confinement preserves AB oscillations even for $L_\varphi^{\mathrm{SS}} \ll L$ (see ref.[4] for details).

In both cases, for very long L_φ^{SS}, the rich content of harmonics in periodic quantum oscillations of the conductance, combined with some frequency shifts due to disorder (see section II A) and to the finite quantum width of surface

states, makes the flux-periodic evolution of the conductance with $B_{\|}$ very different from a pure sine function, as exemplified in Fig. 2. Therefore, at very low temperatures, a detailed study of Aharonov-Bohm oscillations is best done by performing a fast-Fourier transform (FFT) analysis.

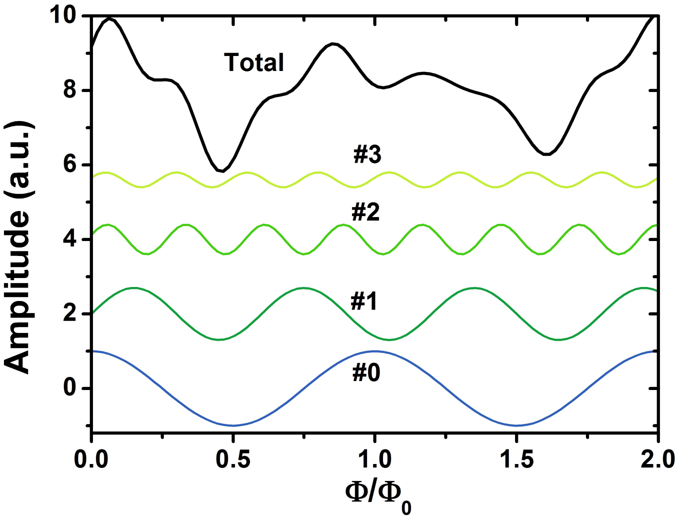

Figure 2: **Non-sinusoidal Aharonov-Bohm oscillations ($L_\varphi^{SS} \gg L_p$).** Contributions of four harmonics to Aharonov-Bohm oscillations altered by disorder (the nth order of each harmonic is indicated after the number sign "♯"). The AB frequency is shifted by +20% for the first harmonic and by −20% for the second harmonic, with respect to the fundamental harmonics $n = 0$, accounting for the typical width of AB peaks in the FFT spectrum observed experimentally. Each harmonic has a different zero-flux phase. As a result, the total contribution (black line) strongly deviates from a pure sine function, and the periodic nature of conductance oscillations is best revealed in the FFT spectrum of a magneto-conductance trace.

B. Influence of a perpendicular field

Applying a perpendicular magnetic field $B_\perp$, both bulk states and topological surface states lead to conductance fluctuations but their nature, as well as their amplitude and correlation field is very different. This is due to a couple of microscopic parameters that are different, such as the enhanced transport length and phase coherence length for topological surface states with respect to bulk states, but also to the diffusive nature of massive quasi-particles whereas the transport of spin-helical Dirac surface modes is quasi-ballistic.

As sketched in Fig. 1, the different quantum corrections to the conductance are:

- Aperiodic *universal* conductance fluctuations from bulk states. Since in general $L_\varphi^{BS} < L$, their amplitude has a power-law dependence with L_φ^{BS}/L, and their relative contribution to conductance fluctuations is reduced by the self average between uncorrelated coherent segments.
- Aperiodic *non-universal* conductance fluctuations from topological surface states. As discussed in this paper, their amplitude depends on both L_φ^{SS}/L and l_{tr}/L, as well as on the energy spectrum and transmissions of quantized transverse modes. Their dominant contribution to conductance fluctuations is a direct consequence of the enhancement of L_φ^{SS}, due to both anisotropic scattering and quantum confinement.

II. EXPERIMENTS

A. Aharonov-Bohm oscillations in 3D topological insulator quantum wires

The periodicity of Aharonov-Bohm oscillations is not necessarily seen in $G(B_{\|})$ traces directly. As shown in Fig. 3, it depends on how the phase coherence length L_φ^{SS} compares to the perimeter L_p. In the narrow Bi_2Se_3 quantum wire considered in this study, $L_\varphi^{SS} \gg L_p$, so that the conductance is modified by the interference of coherent paths corresponding to multiple windings around the perimeter. Aharonov-Bohm oscillations thus have a rich pattern of

harmonics (see ref.[5] for details), the relative amplitude of which depends on disorder. As discussed in section I, $G(B_{\|})$ traces strongly deviate from a pure sine function. Since disorder modifies the relative contribution of Aharonov-Bohm harmonics to the conductance, this effect can be tuned by applying a constant transverse magnetic field, as shown in Fig. 3, Left). In the wider Bi_2Te_3 quantum wire considered in this study, $L_\varphi^{SS} \approx L_p$, so that only the fundamental h/e Aharonov-Bohm harmonic contributes to quantum interference, and periodic oscillations can be directly seen in the conductance. For such a wide nanostructure, the slowly-varying background is not negligible, but periodic quantum oscillations can be easily separated since the Aharonov-Bohm period is small. The influence of disorder on the fundamental harmonic is evidenced by the small phase shifts induced by a transverse magnetic field (see Fig. 3, Right and inset).

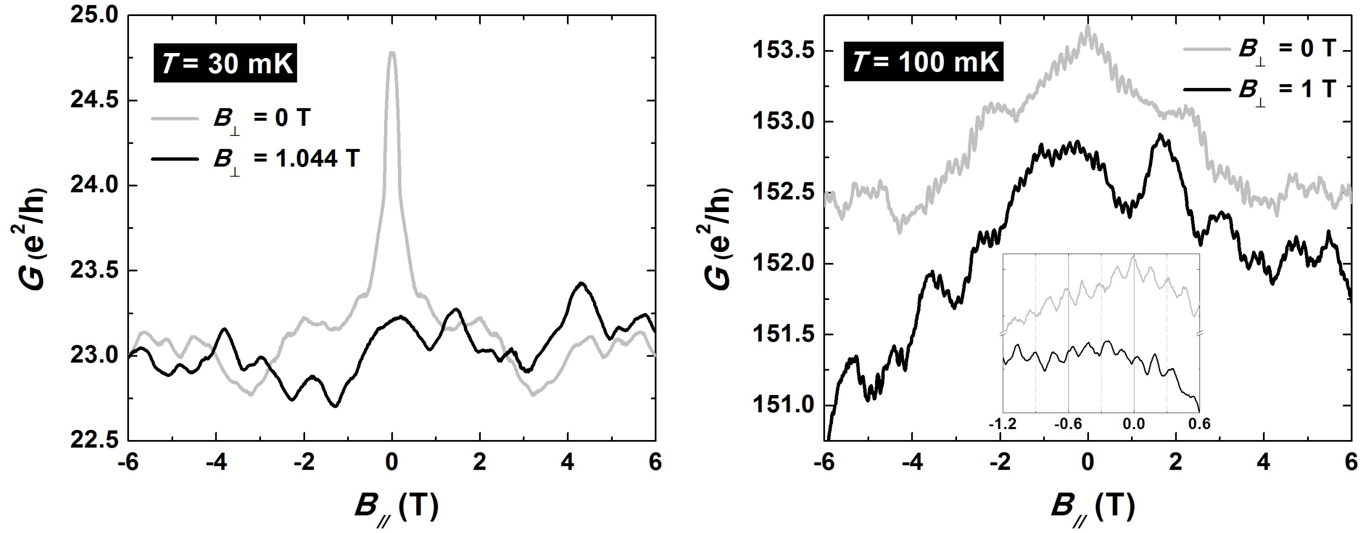

Figure 3: **Influence of a perpendicular magnetic field on Aharonov-Bohm oscillations. Left**, Longitudinal magneto-conductance $G(B_{\|})$ of the narrow Bi_2Se_3 quantum wire ($L_p = 380$ nm), for the length $L_2 = 1$ μm, measured at $T = 30$ mK for two different values of the perpendicular field $B_\perp = 0$ T and $B_\perp = 1.044$ T. **Right**, Longitudinal magneto-conductance $G(B_{\|})$ of the wider Bi_2Te_3 quantum wire ($L_p = 940$ nm), for the length $L_1 = 750$ nm, measured at $T = 100$ mK for two different values of the perpendicular field $B_\perp = 0$ T and $B_\perp = 1$ T. The inset is a zoom, revealing disorder-induced phase shifts.

B. Non-universal conductance fluctuations in a narrow Bi_2Se_3 quantum wire

All mesoscopic conductors studied have a length L that is comparable to or much longer than the transport mean free path l_{tr}, so that the longitudinal motion of helical Dirac fermions is diffusive. As expected from theory for quantum coherent transport in a mesoscopic conductor, the amplitude of conductance fluctuations is reduced in long wires when $L > L_\varphi(T)$, due to averaging between uncorrelated coherent segments. The amplitude of non-universal conductance fluctuations is also reduced when the wire length is increased, but this is already happening for $L_\varphi(T) > L > l_{tr}$. As discussed in the main text for a wire length $L_2/l_{tr} \gtrsim 3$, and as also seen in Fig. 4 ($L_1/l_{tr} \gtrsim 1$) and Fig. 5 ($L_3/l_{tr} \gtrsim 20$), a remarkable property of the modulation found in the standard deviation $\delta G_{rms}(B_{\|})$ is its relatively weak temperature dependence, which does not depend on the wire length. It is determined by the transverse quantization and the condition that the energy level broadening Γ is much smaller than the large transverse energy quantization Δ, even for $L > l_{tr}$. A direct consequence is that the phase coherence length does not directly control the temperature dependence of the modulation of δG_{rms} (see also III D). Besides, this modulation has no more temperature dependence below $T^* \approx 1$ K. Since the disorder broadening Γ of energy levels should be independent of the length of a conductor when $L \geq l_{tr}$, the same crossover occurs when thermal broadening becomes smaller than Γ, independent of the wire length, which corresponds to our observations, as discussed in the main text.

It is also important to remark that the relative change in δG_{rms} is much larger than the conductance change due to the Aharonov-Bohm effect, as clearly seen in Fig. 6 for the wire length $L_2 = 1$ μm, with a relative change of about 10% and 1%, respectively. Furthemore, the amplitude of conductance fluctuations does not scale with the conductance, as expected in the metallic limit (number of modes $N = 80 \gg 1$). For instance, the modulation of δG_{rms} is very large in the range [3T-4T] whereas the conductance does not change much. A thorough analysis is given in section II D, confirming that the amplitude of non-universal conductance fluctuations has no correlation with the conductance.

This result finds a simple explanation in the fact that all opened channels contribute to the surface conductance whereas only a limited number of nearly-opened or nearly-closed channels close to E_F contribute to conductance fluctuations.

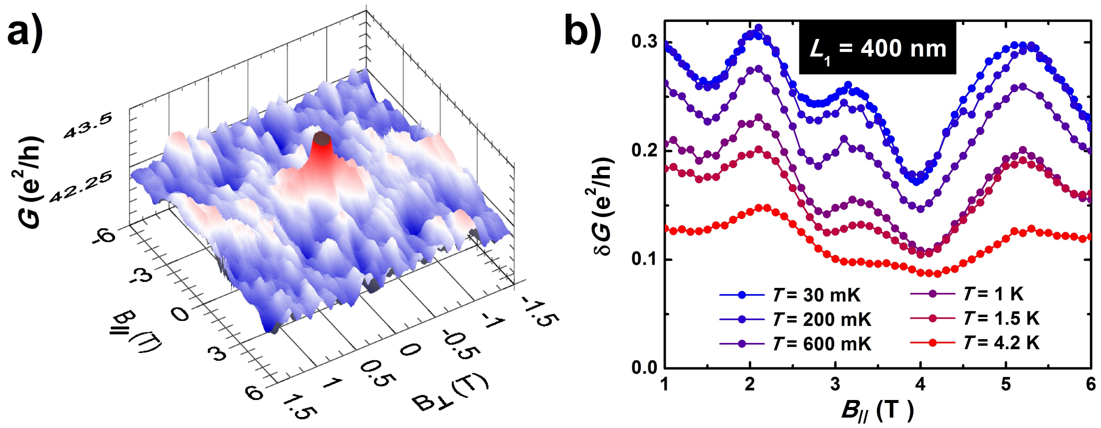

Figure 4: **Non-universal conductance fluctuations in a Bi_2Se_3 quantum wire of length $L_1 = 400$nm, with $L_1/l_{\mathbf{tr}} \gtrsim 1$.** **a**, Mapping of the magneto-conductance measured at T =30mK, showing quantum corrections to the conductance. **b**, Longitudinal-field dependence of δG_{rms}, measured at different temperatures. The modulation of the variance becomes temperature independent below about 1K.

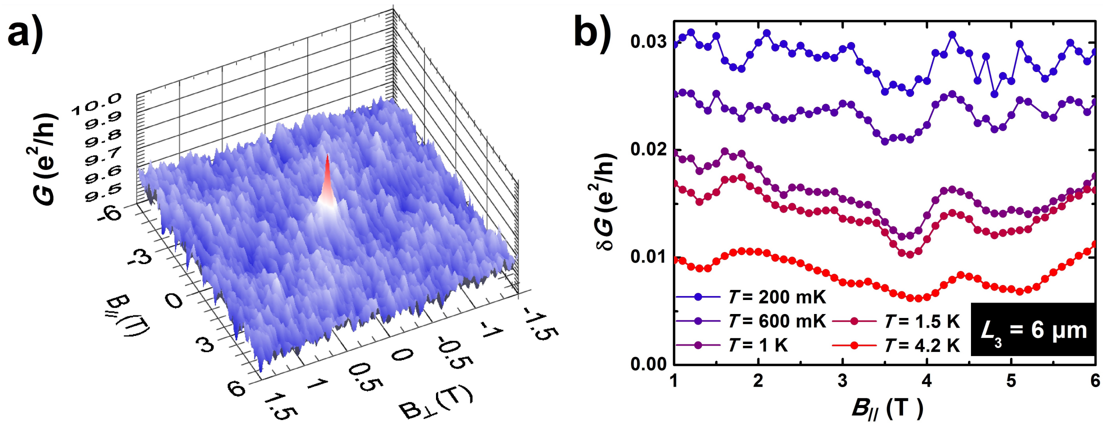

Figure 5: **Non-universal conductance fluctuations in a Bi_2Se_3 quantum wire of length $L_3 = 6$ μm, with $L_3/l_{\mathbf{tr}} \gtrsim 20$.** **a**, Mapping of the magneto-conductance measured at T =200mK, showing quantum corrections to the conductance. **b**, Longitudinal-field dependence of δG_{rms}, measured at different temperatures. The modulation of the variance is strongly damped with respect to shorter wires, but it is still visible and it shows a temperature dependence similar to other wires.

C. Non-universal conductance fluctuations in a wider Bi_2Te_3 quantum wire

Similar results are obtained with Bi_2Te_3 quantum wires. Due to the weaker quantum confinement, the amplitude of non-universal conductance fluctuations is smaller, but still measured with a good accuracy. As shown in the main manuscript for a length $L_1 = 740$ nm, the relative change in δG_{rms} is about 12% whereas it is only 0.1% for the conductance (due to a higher number of opened conduction modes). Moreover, it can be directly seen that there is no correlation between the conductance and the flux-modulation of its variance. The same behavior was found for the longer nanowires, as shown in Fig. 7.

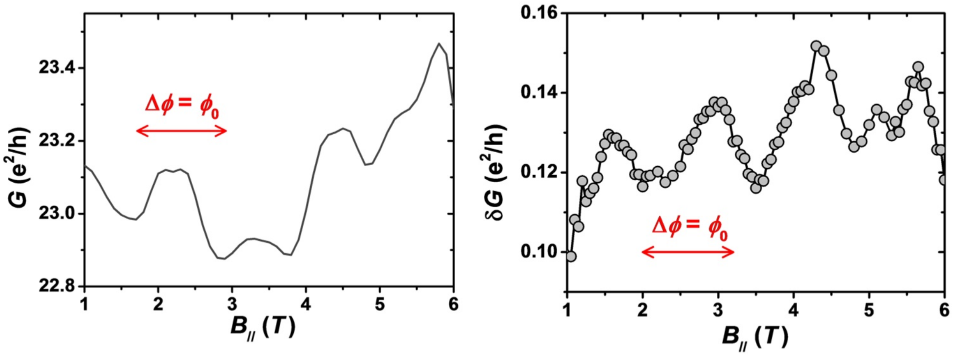

Figure 6: **Non-universal conductance fluctuations in a Bi_2Se_3 quantum wire of length $L_2 = 1$ μm, with $L_2/l_{\mathrm{tr}} \gtrsim 3$.** **Left**, Magneto-conductance in a longitundinal field, measured at base temperature. **Right**, Longitudinal-field dependence of δG_{rms}, inferred from $B_\perp$-sweeps measured in the ± 1.5 T range under a constant $B_\parallel$. The relative change in δG_{rms} is much larger than conductance relative changes. The expected AB period is indicated by red arrows.

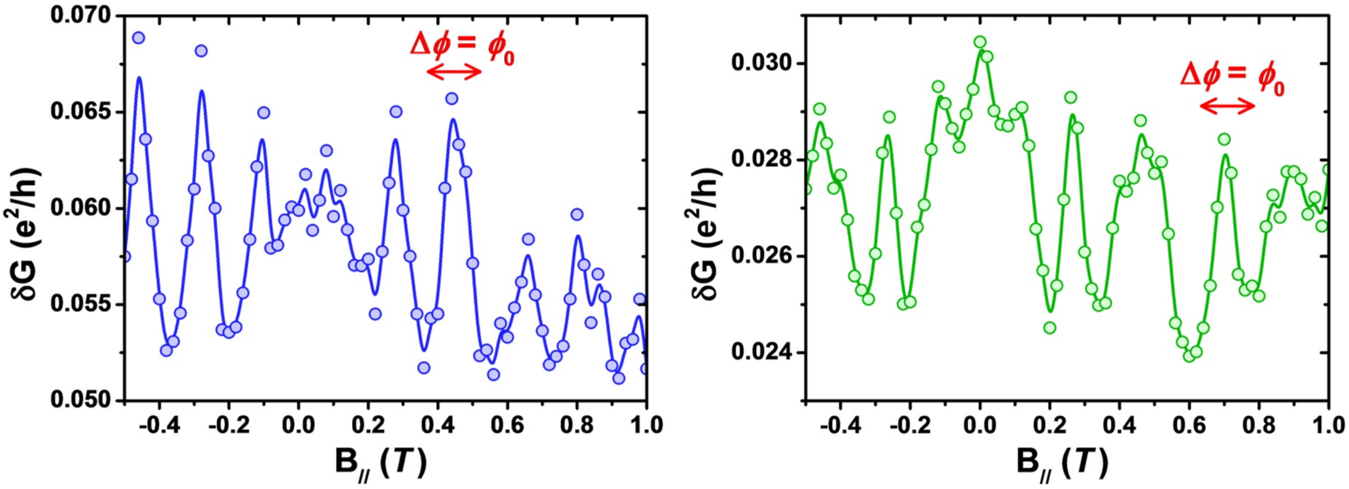

Figure 7: **Non-universal conductance fluctuations in longer Bi_2Te_3 quantum wires.** **Left**, Longitudinal-field dependence of δG_{rms} for a length $L_2 = 1.6$ μm, with $L_2/l_{\mathrm{tr}} \gtrsim 4$. **Right**, Longitudinal-field dependence of δG_{rms} for a length $L_3 = 3.6$ μm, with $L_3/l_{\mathrm{tr}} \gtrsim 9$. Solid lines are B-spline fits corresponding to the data points. The modulation corresponds to the expected Aharonov-Bohm Φ_0 flux period, shown as red arrows.

D. Absence of correlations between the conductance and conductance fluctuations

Similarly to conductance fluctuations, both the mean conductance $\langle G \rangle$ and the conductance at a specific $B_\perp$ (labeled $G_{\mathrm{B}\perp}$ below) depend on $B_\parallel$ and are modulated by the introduction of an Aharonov-Bohm flux. However, and contrary to the case of a ballistic conductor with a small number of conductance channels[7], their is no proportionality between δG_{rms} and $\langle G \rangle$ or $G_{\mathrm{B}\perp}$, as expected for the large number of modes considered here. A simple way to show this is to plot both $\delta G_{\mathrm{rms}}(B_\parallel)$ and $\langle G \rangle (B_\parallel)$ on a full scale. If δG_{rms} would be proportional to $\langle G \rangle$, the relative fluctuations of both quantities $\Delta(\delta G_{\mathrm{rms}})/\overline{\delta G_{\mathrm{rms}}}$ and $\Delta \langle G \rangle / \overline{\langle G \rangle}$, with $\overline{\cdots}$ being the average value over the $B_\parallel$ range measured, should be of the same order of magnitude when changing the flux. This is obviously not the case, as seen in Fig. 6 and in Fig. 8, Left), and all our results give $\Delta(\delta G_{\mathrm{rms}})/\overline{\delta G_{\mathrm{rms}}} \gg \Delta \langle G \rangle / \overline{\langle G \rangle}$, a situation which is specific to weakly-coupled quantized modes.

More generally, we could not find any correlation between δG_{rms} and $\langle G \rangle$ (or $G_{\mathrm{B}\perp}$), as shown in Fig. 8, Right). In this figure, the dotted line refers to the proportionality between δG_{rms} and $\langle G \rangle$ (or $G_{\mathrm{B}\perp}$). Such a scaling can be ruled out, and the large amplitude of the flux-induced modulation of δG_{rms} rather gives a broad vertical line. The zoom-in inset shows the absence of simple correlations between the conductance and its standard deviation. We stress that the size of the “cloud” of reproducible data points is much larger than the error bars shown in the upper right of the inset. In the case of weakly-coupled spin-helical Dirac modes, conductance fluctuations are dominated

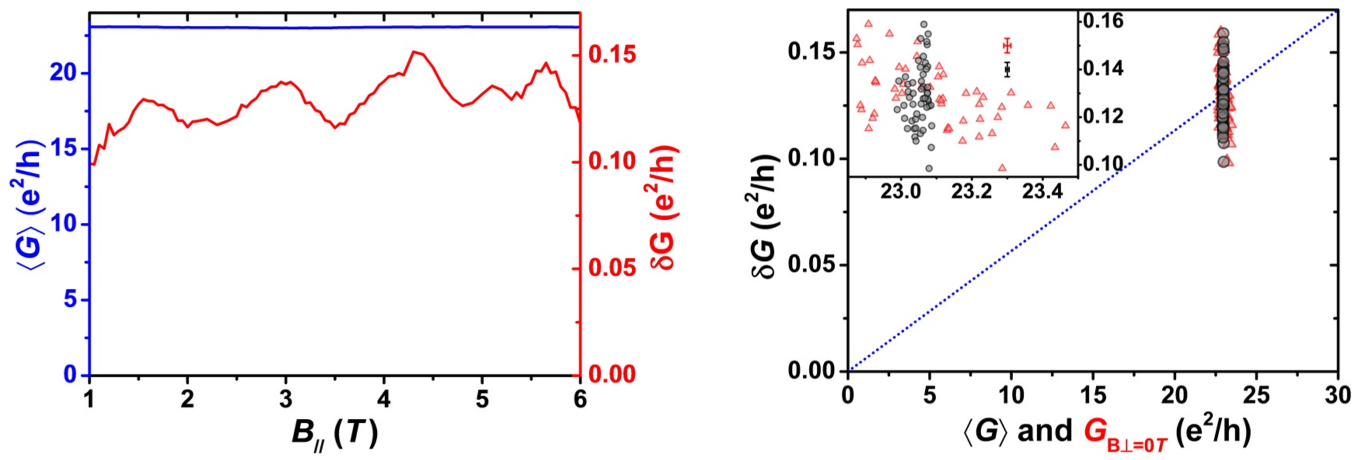

Figure 8: **Absence of correlations between $G(B_\parallel)$ and $\delta G_{\mathbf{rms}}(B_\parallel)$ for the $\mathbf{Bi_2Se_3}$ quantum wire ($L_2 = 1$ μm). Left**, Longitudinal-field dependence of the conductance mean value $\langle G\rangle$ (blue curve) and of the standard deviation of the conductance $\delta G_{\rm rms}$ (red curve) plotted on a full-scale graph, showing the strong difference in their relative variations. **Right**, Amplitude of conductance fluctuations $\delta G_{\rm rms}$ as a function of the conductance mean value (black circles) or of the conductance at $B_\perp = 0$ T (red triangles). The blue dotted line indicates the proportionality between $\delta G_{\rm rms}$ and $\langle G\rangle$ or $G(B_\perp = 0\text{ T})$. Inset : zoom in the data points with error bars indicated in red for $\delta G_{\rm rms}(G(B_\perp = 0\text{ T}))$ and in black for $\delta G_{\rm rms}(\langle G\rangle)$.

only by a small number of opened channels and their amplitude directly depends on the flux dependence of their transmissions, with little correlations with all other propagating modes, whereas the conductance is determined by all opened channels. As a consequence, for a large chemical potential, the relative change in the conductance variance can be much larger than that of the conductance, and an increase of the conductance can nevertheless result in a decrease of its disorder-induced fluctuations.

We also report the same analysis for the results obtained with the Bi_2Te_3 nanoribbon. As seen in Fig. 9, similar conclusions can be drawn, confirming the absence of correlations between the conductance and its variance for our 3D topological insulator quantum wires. As discussed in the main text, our combined experimental and theoretical study reveals that this is a specific property of spin-helical Dirac fermions in presence of quantum confinement, retaining ballistic transport properties despite strong disorder ($L > l_{\rm tr}$) and a high metallicity ($N = E_F/\Delta \gg 1$).

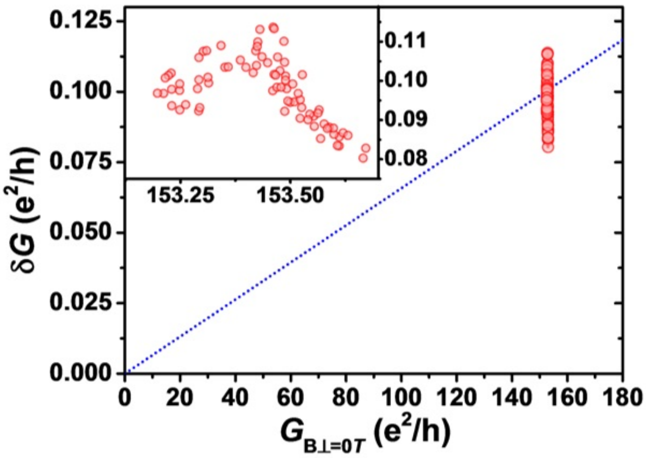

Figure 9: **Absence of correlations between $G(B_\parallel)$ and $\delta G_{\mathbf{rms}}(B_\parallel)$ for the $\mathbf{Bi_2Te_3}$ quantum wire ($L_1 = 740$ nm).** Amplitude of conductance fluctuations $\delta G_{\rm rms}$ as a function of the conductance at $B_\perp = 0$T (red circles). The blue dotted line indicates the proportionality between $\delta G_{\rm rms}$ and $G(B_\perp = 0\text{ T})$. Inset : zoom in the data points.

E. Quantitative estimations of the transport length

To calculate the transport length, different reasonable assumptions about the Fermi energy and the bulk contribution to the conductance of Bi_2Se_3 nanowires have to be made. The values obtained below are based on previous studies we realized with similar nanostructures ([3,8]) and the value of $l_{\rm tr}^{\rm SS}$ used in the main text corresponds to an upper bound. An accurate measurement is made difficult by the finite contact resistance, but realistic values are found in the 150nm-300nm range.

A first method is to infer the value of $l_{\rm tr}$ from the Drude formula in the 2D limit (large number of transverse modes) $G = e^2/h \times \pi \times E/\Delta \times l_{\rm tr}/L$, with L being the length between the contact. This gives a transport length of 185 nm for $L_2 = 1\mu$m and of 135 nm for $L_1 = 400$ nm. Yet, even if relatively small, the contact resistance can significantly influence the estimation of the transport length, due to the rather large conductance of the nanostructures. Taking a typical 150 Ω contact resistance into account, the values of $l_{\rm tr}$ become 255 nm for $L_2 = 1\mu$m and 270 nm for $L_1 = 400$ nm. Also, for such a Fermi energy ($E_{\rm F} \sim 250$ meV is typical for Bi_2Se_3 nanostructures, see[8]), the contribution of bulk carriers to the total conductance cannot be neglected. In the nanowire studied here, it amounts to about half of the total conductance[3] so that the value found above are overestimated. Based the ratio $G_{\rm SS}/G_{\rm bulk} \approx 1.2$ measured in[3], we find $l_{\rm tr} = 140$ nm for $L_2 = 1\mu$m and and $l_{\rm tr} = 150$ nm for $L_1 = 400$ nm.

Another method is based on trans-conductance measurements, as reported in[3], and it gives values close to the low estimations made above. Altogether, an upper bound of 300 nm for $l_{\rm tr}$ is very reasonable, and all mesoscopic conductors studied here satisfy the condition $L > l_{\rm tr}$. A similar analysis for Bi_2Te_3 nanowires gives $l_{\rm tr} < 450$ nm.

III. THEORY

A. Theoretical model

To theoretically model our experiments we adapt a continuous Dirac fermion description of the surface state[4], and take the bulk to be an inert insulator. Although a finite coupling to residual bulk states can increase the scattering of surface states, it remains a small energy that does not modify the energy spectrum of 1D spin-helical surface modes, and therefore does not change the conclusions obtained from our calculations. Explicitly, the surface Hamiltonian reads

$$H = v(\mathbf{p} + e\mathbf{A}) \cdot \sigma + V(\mathbf{r}), \tag{1}$$

where v is the Fermi velocity, $\sigma = (\sigma_x, \sigma_y)$ are Pauli matrices, and the applied magnetic field $\mathbf{B} = \nabla \times \mathbf{A}$. We take $\mathbf{r} = (x, y)$ with x the direction along the length of the wire, and y the periodic transverse direction. The spin of the Dirac fermion is constrained to lie in the tangent plane to the surface, and therefore rotates by 2π going once around the circumference of the wire. This leads to a Berry's phase of π that is taken into account via the boundary condition

$$\psi(x, y + W) = \psi(x, y)e^{i\pi}, \tag{2}$$

with W the wire circumference. Disorder is introduced through the time reversal invariant scalar potential V with correlator

$$\langle V(\mathbf{r})V(\mathbf{r}')\rangle = g(\hbar v)^2/(2\pi\xi^2)e^{-|\mathbf{r}-\mathbf{r}'|^2/2\xi^2}, \tag{3}$$

whereby g is a dimensionless measure of the disorder strength and ξ gives the characteristic length scale of potential variations. The Hamiltonian (1), together with metallic lead boundary conditions, defines a scattering problem that is solved via a transfer matrix technique[9], giving the conductance through the Landauer-Büttiker equation. μ is the chemical potential in the wire.

B. Transmissions of weakly-coupled quantized surfaces modes and Quantum interference

The energy dependence of the transmissions of quantized transverse modes is shown in Fig. 10a, for a flux $\Phi/\Phi_0 = \frac{1}{2}$. The perfectly-transmitted mode ($m = 0$) has a constant transmission equal to one. Despite disorder, the transmissions of higher-energy modes also tend to unity when their longitudinal kinetic energy exceeds their confinement energy, so that the conductance is determined by all opened conduction channels. Small fast oscillations are due to Fabry-Pérot interference between metallic contacts, typical of quasi-ballistic transport, and some resonances are observed

due to disorder (see section III D). An example of quantum corrections to the conductance calculated for a fully-coherent nanowire is shown in Fig. 10b. For a constant transverse field, the magneto-conductance traces $G(B_{\parallel})$ correspond to Aharonov-Bohm oscillations, as shown in Fig. 10c for $B_{\perp} = 1$ T, which result from multi-harmonic interferences for every opened conduction modes. Similar to experiments, their peak-to-peak amplitude is found close to the conductance quantum e^2/h. The nature of magneto-conductance traces $G(B_{\perp})$ is however different. Contrary to Aharonov-Bohm oscillations, which result from all opened modes, the statistics of conductance fluctuations is determined only by a limited number of modes, close to E_F, which are nearly opened or nearly closed. As discussed in the main text, this statistics is not universal and δG_{rms} has a periodic evolution with the flux that is typical of weakly-coupled Dirac fermions in presence of quantum confinement.

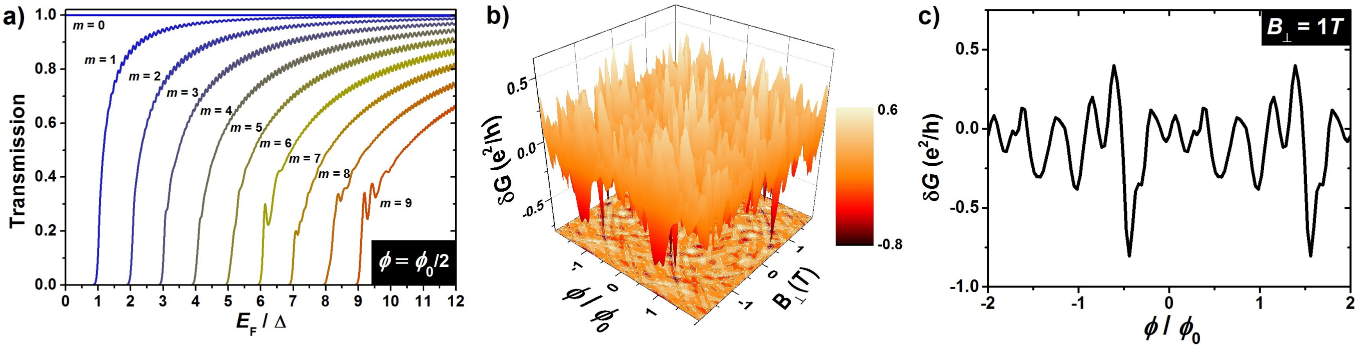

Figure 10: **Transmissions and quantum corrections to the conductance of a disordered 3D TI quantum wire.** **a**, Energy dependence of the transmissions of quantized transverse modes for a flux $\Phi/\Phi_0 = \frac{1}{2}$ (m is the mode index, with the quantized transverse energies $E_m = m\Delta$). **b**, Quantum corrections to the conductance of a disordered conductor with dimensions $w = 120$ nm, $h = 20$ nm and $L = 350$ nm, calculated for an energy $E = 130$ meV and a flux in the $\pm\Phi_0$ range. **c**, Aharonov-Bohm oscillations calculated for a fixed transverse field $B_{\perp} = 1$ T (cross section from b), showing the typical amplitude of quantum interference in the multi-mode transport regime of a disordered 3D TI quantum wire.

C. Transverse-field dependence of the quantized energy spectrum

As previously described in ref.[10], we calculated the energy spectrum of a topological insulator quantum wire with a rectangular cross section (height $h = 20$ nm, width $w = 170$ nm). Fig. 11 shows the dependence of the quantized energy spectrum with a transverse magnetic field $B_{\perp}$. Since a transverse magnetic fields breaks the initial symmetry of the Dirac Hamiltonian, it favors the mixing of transverse modes. Close to the Dirac point, this mixing can be so strong that edge states rapidly develop when $B_{\perp}$ is increased[10], as seen in Fig. 11a). For a fixed value of $B_{\perp}$, the degree of mixing is reduced when the energy of the modes becomes larger than the Zeeman energy. Therefore, $B_{\perp}$ has little influence on the spectrum of high-energy modes and, in the field and energy range studied (see Fig. 11b), the slow increase in the transverse energy remains smaller than the level spacing Δ. Therefore, conductance fluctuations can be studied from $G(B_{\perp})$ traces, independently from the Aharonov-Bohm physics. Besides, the small transverse field-induced change of the conductance cannot explain the large modulation of the conductance variance reported.

D. Transmission of a transverse mode and disorder broadening

Based on our calculations, we show here that the disorder broadening can be deduced from the energy dependence of the transmission $T(E)$ of a transverse mode. As an exemple, we plot below the results obtained for the transmission for the transverse mode $m = 9$ (Fig. 12). Close to the onset energy E_{on}^m of a surface mode, with $E_{\text{on}}^m = (m + 1/2)\Delta$ at $B_{\parallel} = 0$ (that is, for $\Phi_{\text{AB}} = 0$), the transmission is very sensitive to the disorder configuration and it shows a couple of resonances, as shown by vertical arrows in Fig. 12. In this low-energy range, $\partial T/\partial E$ is large, and the modulation of the conductance variance is significant, whereas it decreases at higher energy, so that the nearly opened channel does not contribute to any modulation of the conductance variance anymore. Much smaller oscillations of $T(E)$ seen at higher energies correspond to Fabry-Pérot resonances between metallic contacts.

Importantly, the resonances induced by disorder allow us to estimate the broadening Γ, as inferred from their width at low energy. Whereas a strongly disordered system has broad overlapping resonances, a rather clean system has sharp resonances (and indeed, this case of weak disorder is realized in a disordered 3D topolgical insulator due to anisotropic

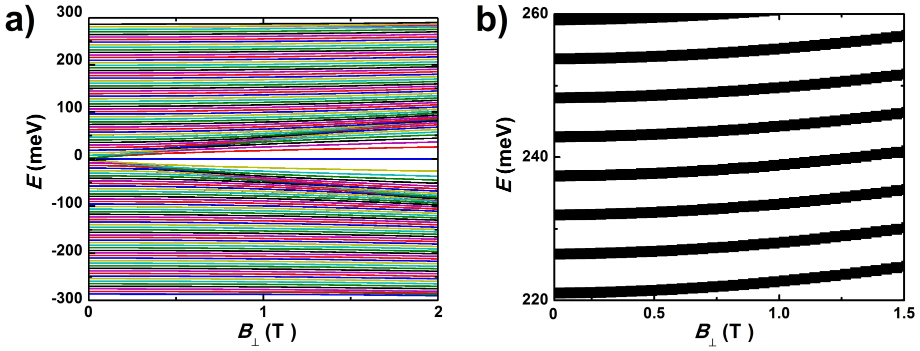

Figure 11: **Influence of a transverse field on quantized modes in a quantum wire.** **a**, Transverse field dependence of the energy spectrum of a nanoribbon with a height $h = 20$ nm and width $w = 170$ nm. **b**, Zoom in the high-energy range, around the value of the Fermi energy for our Bi_2Se_3 quantum wires.

scattering). Still, at high-enough temperatures, the temperature broadening of the Fermi-Dirac distribution ($\sim 4k_{\rm B}T$) further smooths the resonances if $4k_{\rm B}T \gtrsim \Gamma$. The temperature T^* is then defined by $4k_{\rm B}T^* = \Gamma$. From the numerical calculations, we obtain a ratio $\Gamma/\Delta \approx$0.2, a value different but rather close to the experimental result $\Gamma/\Delta \approx$0.06.

Besides, this clarifies the origin of non-universal conductance fluctuations, which result from the nonmonotonous energy dependence of $\partial T/\partial E$ and involve a couple of slightly-opened modes at E_F. Therefore, conductance fluctuations are dominated by the highest energy modes only, and the modulation of the conductance variance is determined by transverse modes with a small kinetic energy (that is, with E_F close to $E^m_{\rm on}$). Near $E_F = 250$meV, this corresponds to three or four partially opened channels. Conductance fluctuations show a maximum when the AB flux is such that $E_{\rm F}$ coincides with the onset of the highest energy mode ($\partial T/\partial E$ is maximum) and they decrease when the AB flux pushes $E_{\rm on}$ away from $E_{\rm F}$ (below or above) so that $\partial T/\partial E$ becomes smaller.

Note that for high-energy transverse modes, a $B_\perp$ sweep is equivalent to an energy scan, since the perpendicular magnetic field induces an overall shift of about $\Delta/2$ in the field range studied. As a result, the fine structure of $T(E)$ for slightly opened modes is well-probed by a $B_\perp$ sweeps, and the non-universal nature of conductance fluctuations affects individual magneto-conductance traces, even for a fixed chemical potential.

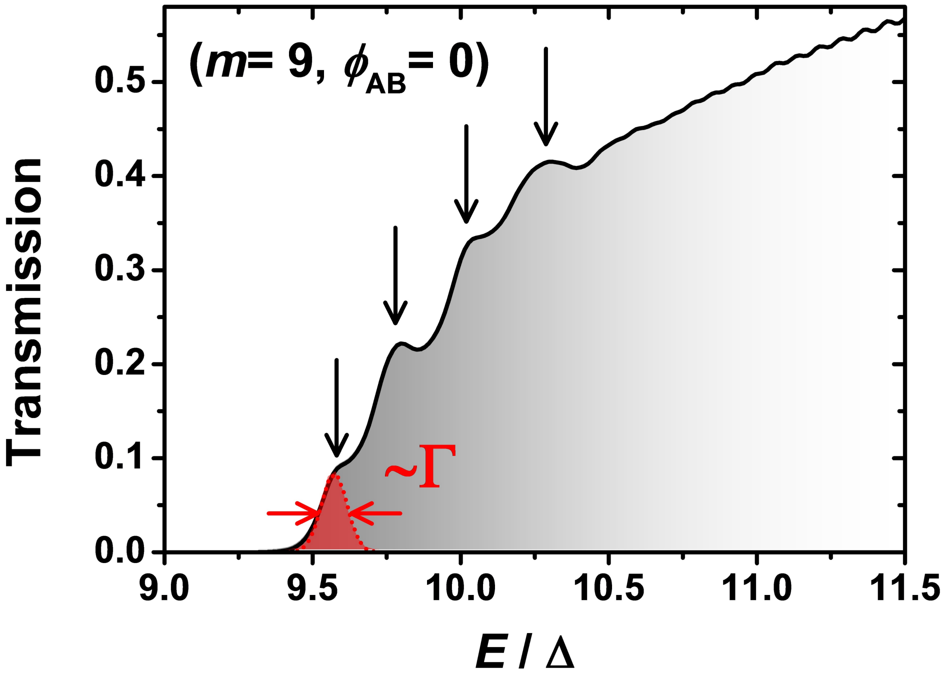

Figure 12: **Calculated transmission of the transverse mode $m = 9$ in zero flux.** Resonances due to disorder are indicated by black arrows. Their width at half maximum roughly corresponds to the broadening Γ (horizontal arrows).

[1] Akkermans, E. and Montambaux, G. *Mesoscopic Physics of Electrons and Photons.* Cambridge University Press, 1 edition, May (2007).

[2] Berry, M. V. *Proceedings of the Royal Society of London A: Mathematical, Physical and Engineering Sciences* **400**, 229–251 (1985).

[3] Dufouleur, J., Veyrat, L., Dassonneville, B., Nowka, C., Hampel, S., Leksin, P., Eichler, B., Schmidt, O. G., Büchner, B., and Giraud, R. *Nano Letters* **16**, 6733-6737 (2016).

[4] Bardarson, J. H., Brouwer, P. W., and Moore, J. E. *Phys. Rev. Lett.* **105**, 156803 Oct (2010).

[5] Dufouleur, J., Veyrat, L., Teichgräber, A., Neuhaus, S., Nowka, C., Hampel, S., Cayssol, J., Schumann, J., Eichler, B., Schmidt, O. G., Büchner, B., and Giraud, R. *Phys. Rev. Lett.* **110**, 186806 Apr (2013).

[6] Jauregui, L. A., Pettes, M. T., Rokhinson, L. P., Shi, L., and Chen, Y. P. *Nat Nano* **11**, 345–351 April (2016).

[7] Marcus, C. M., Rimberg, A. J., Westervelt, R. M., Hopkins, P. F., and Gossard, A. C. *Phys. Rev. Lett.* **69**, 506–509 Jul (1992).

[8] Veyrat, L., Iacovella, F., Dufouleur, J., Nowka, C., Funke, H., Yang, M., Escoffier, W., Goiran, M., Eichler, B., Schmidt, O. G., Büchner, B., Hampel, S., and Giraud, R. *Nano Letters* **15**, 7503–7507 (2015).

[9] Bardarson, J. H., Tworzydło, J., Brouwer, P. W., and Beenakker, C. W. J. *Phys. Rev. Lett.* **99**, 106801 Sep (2007).

[10] de Juan, F., Ilan, R., and Bardarson, J. H. *Phys. Rev. Lett.* **113**, 107003 Sep (2014).
